# Invasive mucormycosis in children: an epidemiologic study in European and non-European countries based on two registries

**DOI:** 10.1186/s12879-016-2005-1

**Published:** 2016-11-10

**Authors:** Zoi Dorothea Pana, Danila Seidel, Anna Skiada, Andreas H. Groll, Georgios Petrikkos, Oliver A. Cornely, Emmanuel Roilides, Collaborators of Zygomyco.net and/or FungiScope™ Registries*, Anupma Kindo, Alberto Arencibia Núñez, Fabianne Carlesse, Jagdish Chander, Cornelia Lass-Flörl, Bertrand Dupont, Werner Heinz, Nikolay Klimko, Sofya Khostelidi, Katrien Lagrou, Livio Pagano, Zdenek Racil, Monika Rolencova, Petr Sedlacek, Vanda Chrenkova, Julia Horakova, Peter Mudry, Maria JGT Vehreschild, Stefan Zimmerli

**Affiliations:** 1 3rd Department of Paediatrics, Infectious Diseases Unit, Aristotle University School of Medicine, Hippokration General Hospital, Konstantinoupoleos 49, GR-546 42 Thessaloniki, Greece; 2 Department of Internal Medicine, Clinical Trials Centre Cologne, ZKS Köln, Center for Integrated Oncology CIO Köln Bonn, Cologne Excellence Cluster on Cellular Stress Responses in Aging-Associated Diseases (CECAD), German Centre for Infection Research, University of Cologne, Cologne, Germany; 3 1st Department of Medicine, Laikon General Hospital, National and Kapodistrian University of Athens, Athens, Greece; 4 Center for Bone Marrow Transplantation and Department of Paediatric Hematology and Oncology, Infectious Disease Research Program, University Children’s Hospital, Muenster, Germany; 5 European University Cyprus School of Medicine, Engomi-Nicosia, Cyprus

**Keywords:** Mucormycosis, Zygomycosis, Paediatric invasive fungal diseases, FungiScope™, Zygomyco.net

## Abstract

**Background:**

Mucormycosis has emerged as a rare but frequently fatal invasive fungal disease. Current knowledge on paediatric mucormycosis is based on case reports and small series reported over several decades. Contemporary data on a large cohort of patients is lacking.

**Methods:**

Two large international registries (Zygomyco.net and FungiScope™) were searched for mucormycosis cases in ≤19 year-old patients. Cases enrolled between 2005 and 2014 were extracted, and dual entries in the two databases merged. Epidemiology, clinical characteristics, diagnostic procedures, therapeutic management and final outcome were recorded and analysed with SPSS v.12.

**Results:**

Sixty-three unique cases (44 proven and 19 probable) were enrolled from 15 countries (54 in European and 9 in non-European countries). Median age was 13 years [Interquartile Range (IQR) 7.7] with a slight predominance (54.1 %) of females. Underlying conditions were haematological malignancies (46 %), other malignancies (6.3 %), haematopoietic stem cell transplantation (15.9 %), solid organ transplantation, trauma/surgery and diabetes mellitus (4.8 % each) and a variety of other diseases (7.9 %); in 9.5%, no underlying medical condition was found. Neutropenia was recorded in 46 % of the patients. The main sites of infection were lungs (19 %), skin and soft tissues (19 %), paranasal sinus/sino-orbital region (15.8 %) and rhino-cerebral region (7.9 %). Disseminated infection was present in 38.1 %. Mucormycosis diagnosis was based on several combinations of methods; culture combined with histology was performed in 31 cases (49.2 %). Fungal isolates included *Rhizopus* spp. (39.7 %), *Lichtheimia* spp*.* (17.5 %), *Mucor* spp. (12.7 %), *Cunninghamella bertholletiae* (6.3 %) and unspecified (23.8 %). Treatment comprised amphotericin B (AmB) monotherapy in 31.7 % or AmB in combination with other antifungals in 47.7 % of the cases, while 14.3 % received no antifungals. Surgery alone was performed in 6.3 %, and combined with antifungal therapy in 47.6 %. Crude mortality at last contact of follow-up was 33.3 %. In regression analysis, disseminated disease and prior haematopoietic stem cell transplantation were associated with increased odds of death, whereas the combination of systemic antifungal therapy with surgery was associated with improved survival.

**Conclusion:**

Paediatric mucormycosis mainly affects children with malignancies, presents as pulmonary, soft tissue, paranasal sinus or disseminated disease and is highly lethal. Outcome is improved when active antifungal therapy and surgery are combined.

**Electronic supplementary material:**

The online version of this article (doi:10.1186/s12879-016-2005-1) contains supplementary material, which is available to authorized users.

## Background

Mucormycosis in children has emerged as an increasingly important infection and is associated with significant morbidity and mortality [1, 2]. Mucorales are generally characterized by their tendency for rapid vascular invasion leading to vascular thrombosis and tissue necrosis. As a result, the Mucorales may initially cause localized infections, but they rapidly progress to invasive soft tissue, rhinocerebral, orbital, gastrointestinal or pulmonary infections [2, 3].

Risk factors for mucormycosis in adults include profound and prolonged neutropenia, hematological malignancies, bone marrow or solid organ transplantation, uncontrolled diabetes mellitus with metabolic acidosis, iron overload, deferoxamine therapy, previous trauma, whereas in few cases no apparent underlying condition can be identified [4–10]. In paediatric reviews, prematurity has also been a risk factor for invasive mucormycosis, and age <12 months was an independent risk factor for mortality [1, 11, 12]. While some investigators have shown an increasing incidence in adults [13], the epidemiological data in children are too limited as to provide epidemiological trends [2]. Due to the rarity of the disease and the limited awareness of physicians to diagnose and record these cases, the true burden in the paediatric setting is difficult to be estimated.

The aim of this study was to collect and analyse data of paediatric mucormycosis cases from two international case registry databases to update and broaden our knowledge on epidemiology, clinical characteristics, diagnostic procedures, microbiology, treatment and outcome of mucormycosis in children.

## Methods

### Study design

The study was a combined analysis of prospectively collected cases enrolled in two international invasive fungal disease (IFD) registries: Zygomyco.net and FungiScope™. Both databases were searched for invasive infections due to Mucorales in paediatric (≤19 years) patients. Cases diagnosed between 2005 and 2014 were extracted from the registries and analysed. The selection of the study period was based on the availability of data in the two registries.

Zygomyco.net was created in 2004 as a European registry and later became a global registry for invasive mucormycosis of the European Confederation of Medical Mycology/International Society for Human and Animal Mycology (ECMM/ISHAM) Working Group on Zygomycosis. For this database, a national coordinator in each participating European country was appointed to prospectively collect and record confirmed cases in standardized case report forms (CRFs) at www.zygomyco.net, which were then sent either by e-mail or fax to the general study coordinator (GP). The national coordinators were internationally recognized experts in the field of fungal infections, and in most cases they were appointed by the respective national mycology societies. Diagnosis of cases was based on standard mycological tools and when isolate was available by molecular methods in two central laboratories [9].

FungiScope™ – Global Emerging Fungal Infection Registry (ClinicalTrials.gov NCT01731353) was created in 2003 as an international university-based case registry that collects data of patients with rare IFD, using a web-based electronic case form at www.fungiscope.net. The survey was hosted by ClinicalSurveys.net, an internet-based survey software.

For both databases case registration was on a voluntary, non-systematic basis. Data sets were submitted in a pseudonymized form and regularly checked for completeness with queries made in written form for missing data. Follow-up information was repeatedly requested regarding treatment response, survival or death. If follow-up could not be continued at all requests, outcome at last contact was used.

The final analysis of the dataset was performed after checking for and deleting overlapping cases enrolled in both registries. The final combined database included the following variables: gender, age, diagnostic procedures for documenting infection, anatomic location, underlying conditions, microbiology, management and outcome.

### Definitions

The term mucormycosis was used in this study as a synonym of zygomycosis [14]. Mucorales are one of the two orders comprising Zygomycetes, that is Mucorales and Entomophthorales [14]. In particular, Mucorales consist of genera including *Rhizopus, Mucor, Lichtheimia (Absidia), Cunninghamella* and *Saksenaea*.

For the present study, a paediatric patient was defined as an individual ≤19 years of age.

The day of diagnosis was defined as the day on which the first diagnostic procedure identifying a Mucorales was performed. For patients with a diagnosis obtained during post-mortem examination, the day of death was considered to be the day of diagnosis.

#### Classification of infection

The sites of infection were classified according to those utilized in the study of Roden et al*.* [15]*.* Accordingly, dissemination was defined as two or more non-contiguous locations of Mucorales infection; sino-orbital disease as involvement of the paranasal sinus and orbit, without extension to the brain; pulmonary infection as localised when confined to lung tissue and as deep extension when invading adjacent tissues. Cutaneous infection was characterized as localised when it did not extend to underlying tissues, in which case it was characterized as having localized soft tissue infection.

For the classification of each case as proven or probable, the revised definitions of IFD of the European Organization for Research and Treatment of Cancer/Mycosis Study Group (EORTC/MSG) were used [16]. Proven mucormycosis was defined when both histology and culture [or polymerase chain reaction (PCR)] existed and probable mucormycosis when only histology (proven IFD) or positive culture from a normally non-sterile site.

Outcome was defined as survival or mortality at last contact of follow-up. Mortality was assessed as all-cause mortality during the course of mucormycosis.

### Statistical analysis

Statistical analysis was conducted using SPSS v.12. Differences between the qualitative variables in two or more groups were analysed by chi-square test. A two-sided p value of <0.05 was considered significant. For the estimation of predictors of outcome univariate analysis was performed using logistic regression for each variable separately. The variables for which a statistically significant relationship was shown (p <0.05) were used to construct a new multivariate model using the logistic regression approach. In the final regression analysis lost-to-follow up cases were excluded.

### Ethics

Recording patients’ data in the zygomyco.net and analysing them was approved by the Ethics Committee of the National and Kapodistrian University Attikon General Hospital located in Athens, Greece, the institution of the registry principal investigator (reference 9/30-8-2011). Informed consent was obtained if required by local laws or regulations. Ethics Committees that approved data collection and use for this registry are in Additional file 1.

Similarly, to record patients’ data in FungiScope™ informed consent was obtained if required by local laws or regulations. The ethics committee at University of Cologne, Germany, confirmed the data protection and privacy policy (reference-ID 05-102) [17]. Ethics Committees that approved the protocol for data collection and use in the Fungiscope registry are in Additional file 2.

## Results

### Demographics

**Fig. 1:**
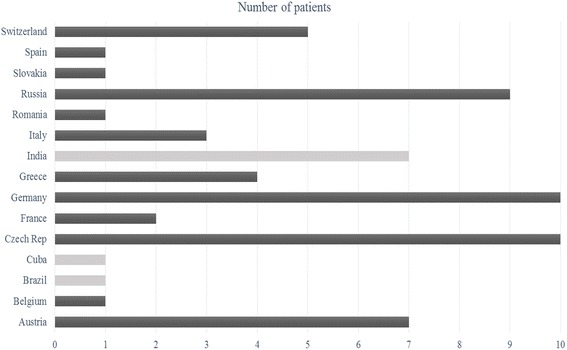
The contribution of European and non-European countries in the paediatric mucormycosis cases of the two registries studied. The names of the countries are shown in alphabetical order. Dark grey columns represent European countries (EU) while light grey colours represent the non-EU ones

During the period 2005–2014, 63 unique paediatric mucormycosis cases eligible for inclusion were submitted to one or both registries from 15 countries. Thirty-six were registered in Zygomyco.net, 17 in FungiScope™, whereas 10 were registered in both registries. Of the total of 63 cases, 54 cases (85.7 %) were from European (EU) countries, while 9 cases (14.2 %) were from non-European countries (non-EU) (Fig. 1). The median age of the patients was 13 years [Interquartile Range (IQR) 7.7] with a slight predominance of female gender (54.1 %). Age of ≤1 year accounted for 7.9 % of the study population (5 cases). The median age among EU and non-EU cases did not show significant difference (13 years vs. 15 years, respectively; p = 0.51).

### Underlying conditions

The patients’ underlying conditions are listed in Table 1. The majority of children had an underlying haematological malignancy (29 patients, 46 %). Among them, acute lymphoblastic leukaemia (ALL) was reported in 16 cases, acute myeloid leukaemia (AML) in 11 cases, and non-Hodgkin lymphoma (T-NHL) as well as myelodysplastic syndrome in one case each. Other malignancies were recorded in four cases (6.3 %): brain tumors in two cases, and osteosarcoma and thoracic carcinoma in one case each. At the time of diagnosis, ten patients (15.9 %) and three patients (4.8 %) had undergone haematopoietic stem cell (HSCT) or solid organ transplantation (SOT), respectively. Two of the solid organ transplants included lung. Other underlying conditions (7.9 %) were two cases of aplastic anaemia and one case each Pearson’s syndrome [18], systemic lupus erythematosus and asplenia. The child with Pearson’s syndrome underwent therapy with deferoxamine and methylprednisolone on diagnosis. Diabetes mellitus (DM) alone was present in three patients (4.8 %), while one patient with T-NHL presented also a steroid-induced DM at the time of diagnosis. Previous traumatic injury or surgery was recorded in three patients (4.8 %). In one case trauma was induced by a car accident, while surgery was reconstructive Fallot surgery in two cases. In the remaining six patients (9.5 %) no underlying medical condition was found. Neutropenia was reported in 29 (46 %) patients. The representation of the underlying diseases differed significantly among EU and non-EU mucormycosis cases (p = 0.01). The most common underlying disease for the EU cases was haematological malignancies (35/54; 64.8 %), while for the majority of non-EU cases no underlying condition was reported (5/9; 55.5 %).

**Table 1:** Underlying conditions and associated crude mortality rates in 63 paediatric patients with invasive mucormycosis

Underlying conditions	Cases (%)	Mortality (%)^f^
Haematological malignancies^a^	29 (46)	9/23 (39.1)
HSCT^b^	10 (15.9)	8/10 (80)
SOT^c^	3 (4.8)	2/2
Other malignancies^d^	4 (6.3)	0/3
Diabetes mellitus	3 (4.8)	0/2
Other conditions^e^	5 (7.9)	0/4
Trauma/surgery	3 (4.8)	1/3
None	6 (9.5)	1/6

^a^ Acute lymphoblastic leukaemia (ALL), acute myeloid leukaemia (AML), Hodgkin lymphoma (HL), non-Hodgkin lymphoma (NHL), myelodysplastic syndrome

^b^ haematopoietic stem cell transplantation

^c^ solid organ transplantation

^d^ Osteosarcoma, thoracic and brain tumour

^e^ Hyposplenism, Pearson syndrome, aplastic anaemia, systemic lupus erythematosus

^f^ In 10 patients (15.8%) mortality rates were not recorded due to lost to follow up

### Sites of infection

The anatomic sites of infections and the localization of infection according to the main underlying condition in the 63 cases are listed in Table 2. Disseminated infection at the time of diagnosis was found in 24 (38.1 %) cases. Dissemination to more than three sites was described in four cases. Gastrointestinal infection was reported as part of disseminated disease in nine cases. Other reported locations were pulmonary (localized or with deep extension) and cutaneous or localized soft tissue infections in 12 (19%) cases each, followed by paranasal sinus plus sino-orbital in 10 (15.9%) and rhinocerebral in 5 (7.9 %) cases, respectively. Haematological malignancy correlated with pulmonary disease; among 29 patients with pulmonary focus (alone or part of disseminated disease), 22 had a haematological malignancy [75.8 %; p = 0.01; Odds Ratio (OR) = 4.4, 95 % Confidence Intervals (CI) = 1.5-13.3]. Diabetes did not correlate with rhinocerebral disease, although the number of patients with DM was small (3 patients). A significant difference between the site of infection and the origin of cases was observed. In particular, the most common site of infection for the EU cases was disseminated disease (22/54; 40.7 %), while for the non-EU cases paranasal sinus plus sino-orbital site (5/9; 55.5 %) was most frequently reported (p = 0.007).

**Table 2:** Sites of infection according to the underlying conditions in 63 paediatric cases of mucormycosis

Underlying conditions	Dissemination	Pulmonary/deep extension	Cutaneous + soft tissue	Sinus + sinoorbital	Rhinocerebral
Haematological malignancies	12/29 (41.3^a^)	7/29 (24.1)	4/29 (13.7)	4/29 (13.7)	2/29 (6.8)
HSCT^b^	6/10 (60)	3/10 (30)	1/10 (10)	0	0
SOT	1/3 (33.3)	1/3 (33.3)	1/3 (33.3)	0	0
Other malignancies	2/4 (50)	1/4 (25)	0	0	1/4 (25)
Diabetes mellitus	0	0	1/3 (33.3)	1/3 (33.3)	1/3 (33.3)
Trauma/surgery	1/3 (33.3)	0	2/3 (66.6)	0	0
Other conditions	1/5 (20)	0	2/5 (40)	1/5 (20)	1/5 (20)
None	1/6 (16.6)	0	1/6 (16.6)	4/6 (66.6)	0
Total					
Number of cases (%)	24 (38.1)	12 (19)	12 (19)	10 (15.9)	5 (7.9)
Mortality (%)	13/21 (61.9)	3/10 (30)	0/9 (0)	4/10 (40)	1/3 (33.3)

*HSCT* hematopoietic stem cell transplantation, *SOT* solid organ transplantation

^a^ Numbers in parenthesis are percentages

^b^ In 10 patients (15.8%) mortality rates were not recorded due to lost to follow up

Dissemination was defined as >1 non-contiguous locations of Mucorales infection

Pulmonary infection was characterized as localized when it was only in the lung tissue and with deep extension when it extended to adjacent tissues, such as pleura and heart

Sino-orbital refers to cases involving sinus and orbit, without extension to brain

### Diagnosis

Diagnostic criteria for mucormycosis are listed in Table 3, and the causative pathogens are shown in Table 4. In total, 11 different diagnostic methods or combinations of methods were used for the documentation of mucormycosis. In particular, diagnosis was made only by culture in 11 patients (17.5 %) and by histology in seven patients (11.1 %); both were positive in 12 patients (19 %). Fungal PCR in addition to culture, histology or direct microscopy was performed in 11 cases (17.5 %). The combination of direct microscopy, histology and culture was reported in 12 cases (19%). In two cases the diagnosis of mucormycosis was made post-mortem at autopsy. Among the 63 pediatric patients enrolled, 44 (69.8 %) were classified as proven mucormycosis cases and the remaining (30.2 %) as probable. The most frequently recorded species for EU and non–EU countries were *Rhizopus* spp. (26 cases, 46.3 %), followed by *Lichtheimia* spp. (10 cases, 15.9 %). In 15 patients (23.8 %) Mucorales remained unspecified.

**Table 3:** Various diagnostic methods of paediatric mucormycosis cases

Diagnostic method(s) used	Cases (%)
Culture	11 (17.5)
Histology	7 (11.1)
Direct microscopy	1 (1.6)
Histology + direct microscopy	6 (9.5)
Histology + culture	12 (19)
Direct microscopy + culture	3 (4.8)
PCR^a^ + histology	2 (3.2)
PCR + culture	2 (3.2)
Direct Microscopy + Histology + Culture	12 (19)
Histology + culture + PCR	3 (4.8)
Direct Microscopy + Histology + Culture + PCR	4 (6.3)

^a^ PCR: polymerase chain reaction

**Table 4:** Causative pathogens identified in 63 pediatric patients with invasive mucormycosis

Pathogen	Cases (%)	Mortality (%)
*Rhizopus* spp.*:*	26 (41.3)	11/24 (45.8)
*R. oryzae*	8	3/7 (42.8)
*R. microsporus*	4	3/4 (75)
*R. homothallicus*	1	0/1
Unspeciated	13	5/13 (38.4%)
*Lichtheimia* spp.*:*	10 (15.9)	4/8 (50)
*L. corymbifera*	7	2/6 (33.3)
*L. ramosa*	2	1/2 (50)
Unspeciated	1	1/1 (100)
*Mucor* spp.	8 (12.7)	0/7 (0)
*Cunninghamella* spp.	4 (6.3)	1/3 (33.3)
Unidentified Mucorales	15 (23.8)	5/11 (45.4)

### Therapy and outcome

The treatment of mucormycosis is outlined in Table 5. Of the 63 patients included in the study, four patients (6.3 %) received no treatment either because diagnosis was made post-mortem or patients were lost to follow-up. Mucormycosis was treated by surgery alone in four patients (6.3 %). It had successful outcome in two patients with localized skin and soft tissue infection, and dismal outcome in two patients with disseminated disease. In total, 25 patients (39.7 %) received systemic antifungal therapy without surgery. Another 30 patients (47.6 %) received antifungal therapy and surgery. In patients receiving antifungal therapy, AmB was the first line treatment given either alone or in combination with other antifungal agents as shown in Table 6. Monotherapy with AmB was recorded in 20 (31.7 %) patients. AmB plus caspofungin was administered in 4 (6.3 %) patients and AmB plus micafungin in one patient. AmB plus posaconazole was administered in 14 patients (22.2 %).

**Table 5:** Treatment and outcome of 63 paediatric patients with invasive mucormycosis

Treatment	Cases (%)	Mortality (%)^a^
Surgery and antifungal drugs	30 (47.6)	5/27 (18.5)
Only antifungal treatment	25 (39.7)	12/20 (60)
Only surgery	4 (6.3)	2/4 (50)
No treatment	4 (6.3)	2/2 (100)

^a^ In 10 patients (15.8%) mortality rates were not recorded due to lost to follow up

**Table 6:** Antifungal drug treatments with or without surgery

Antifungal treatment	Cases (%)	Mortality (%)
AmB	20 (31.7)	4/17 (23.5)
AmB + POS^a^	14 (22.2)	3/14 (21.4)
AmB + CAS^b^	4 (6.3)	2/3 (66.6)
AmB + VORI^c^ + CAS + POS	2 (3.2)	0/1
AmB + CAS + FLU^d^	1 (1.6)	1/1
CAS	2 (3.2)	1/1
CAS + VORI	2 (3.2)	2/2
AmB + MIC^e^	1 (1.6)	0/1
AmB + VORI	1 (1.6)	0/1
POS + FLU + CAS	1 (1.6)	1/1
POS	1 (1.6)	not reported, NR

^a^POS: posaconazole; ^b^CAS: caspofungin; ^c^VORI: voriconazole; ^d^FLU: fluconazole; ^e^MIC: micafungin

Overall mortality was 33.3 %. In 10 (15.8 %) children mortality was not recorded because they were lost to follow up. Mortality did not differ significantly among EU and non-EU cases (19/45; 42.2 % versus 2/18; 25 %, p = 0.45). Mortality rates were variable depending on the underlying condition of the patients, the site of infection, and treatment administered (Tables 7 and 8). The risk factors affecting mortality on univariate analysis were HSCT, disseminated disease and administration of antifungal drugs alone, while combined treatment was protective. On multivariate analysis, HSCT and dissemination remained independent predictors of death.

**Table 7:** Univariate analysis of risk factors for mortality

Variables	Death rate (%)	*p-value*	OR (95% CI)^a^
Female gender	11/28 (39.2)	0.991	
Age < 5 years	3/10 (30)	0.687	
Haematological disorder	15/31 (48.3)	0.121	
AML^b^	5/11 (45.4)	0.736	
HSCT^c^	8/10 (80)	0.009	2.6 (1.5–4.5)
Neutropenia	13/26 (50)	0.166	
No underlying condition	1/6 (16.6)	0.384	
Dissemination	13/21 (61)	0.0085	2.47 (1.2–4.9)
Trauma/Surgery	1/3 (33.3)	0.819	
Combined treatment	5/27 (18.5)	0.002	0.3 (0.12–0.7)
Only antifungal treatment	11/19 (57.8)	0.042	1.9 (1–3.7)

^a^ OD: Odds Ratio; CI: Confidence intervals

^b^ AML: Acute myeloid leukemia

^c^ HSCT: Hematopoietic stem cell transplantation

**Table 8:** Multivariate analysis of risk factors for mortality

Variables	*p-value*	OR (95% CI)^a^
HSCT^b^	0.01	13.66 (1.88–98.9)
Only antifungal treatment	0.271	2.3 (0.5–10.6)
Combined treatment	0.035	0.37 (0.1–0.9)
Disseminated infection	0.05	4.2 (0.9–18.5)

^a^ OD: Odds Ratio; CI: Confidence intervals

^b^ HSCT: Hematopoietic stem cell transplantation

## Discussion

This is the largest multinational analysis of contemporary data of paediatric mucormycosis cases based on electronic recording in two independent global registries. Based on the results of the present study, mucormycosis in children seems to be a rather heterogeneous infectious disease as far as mortality rate is concerned. This is directly associated with the dissemination of the infection and the underlying condition of the patients. In particular, although the crude mortality rate for the whole paediatric cohort was 33.3 %, the percentage of children who died with disseminated mucormycosis was significantly higher (61 %) compared to the children with localized disease (0 %). Furthermore, in children suffering from malignancies the mortality rate ranged from 41.3 % to 66.6 %, while in children without any underlying condition the mortality rate was relatively low (16.6 %). The multivariate analysis confirmed the observations of the initial descriptive analysis revealing that two of the main independent significant factors influencing mortality in paediatric mucormycosis were disseminated infection and HSCT. These results emphasize the need for high awareness and prompt diagnosis especially during severe immunocompromise. Mucorales rapidly invade vessels and disseminate, thus antifungal therapy combined with surgical debridement significantly impacted on mortality in our multivariate analysis. Irrespective from other confounding variables, children receiving antifungal therapy and surgery had a mortality rate of 18.5 %, while patients receiving antifungal therapy alone or no therapy at all reached a mortality rate of 60 % and 100 %, respectively. The abovementioned results should be interpreted with caution since the rather small sample size of the children precluded weighing for potential confounding factors.

Haematological malignancies and HSCT predominated as underlying conditions. DM was present in only 5 %, while in 10 % of the children no predisposing factor was identified. The predominance of immunocompromised patients could be explained either by increase of the frequency of such children worldwide or by reporting bias and increased awareness of haematology centers to participate and include cases in registries. In the largest subgroup of immunocompromised children, as depicted in Table 2, disseminated and pulmonary infection were found, in concordance with previous literature, as the most frequent sites of infection. By comparison, localized skin and soft tissue infections or sinus infections were more frequently detected in cases associated with trauma/surgery or without any underlying condition [1, 2, 9, 10, 19]. DM was not associated with rhinocerebral mucormycosis possibly due to the small number of DM cases [9, 15, 19]. In a previous review article, gastrointestinal (GI) infections accounted for almost 20 % of children with mucormycosis, but the majority of these patients were neonates, in which the final diagnosis was placed post-mortem [1]. This might have led to underrepresentation of this patients’ group in the registries of the present study. However, we observed few GI infections recorded as part of disseminated disease, possibly due to delay in diagnosis of mucormycosis. Additionally, the median age in the review [1] was 5 years with a respectable number of infants, while the median age in our registry study was 13 years with a small proportion of cases below one year of age (8 %). Irrespectively of the exact percentages of underlying conditions it is important to emphasize that infections due to Mucorales are observed in rather heterogenic paediatric populations with a wide spectrum of organ involvement and subsequently with significant differences in treatment approach and outcome.

The distribution of Mucorales revealed that *Rhizopus* spp. remains the most frequent species isolated in children, followed by *Lichtheimia* spp. Of interest, in a previous systematic review of published paediatric cases, the second most frequent specie isolated was *Mucor* sp. [1]. According to a recent epidemiological study by Lanternier et al. the prevalence of *Lichtheimia* spp. in Europe ranges from 18 % to 24 %, while in similar studies from Asia and the US *Lichtheimia* spp. are recorded with a frequency of <10 % [19]. In our study, the majority of data derived from European countries (54 cases) and the percentage of *Lichtheimia* spp. recorded was almost 16%. Based on this information, the percentage of *Lichtheimia* spp. in our study may not directly reflect an actual epidemiological rise of these species in children. However, it may represent different fungal biogeography distribution among European and non-European countries that needs further confirmation with extended analysis of the local fungal niches worldwide.

Treatment options against mucormycosis consisted mainly of combined therapy (antifungal therapy and surgery) recorded in 47.6 % or of antifungal therapy alone reported in almost 40 % [9, 20]. Amphotericin B was the drug of choice given either as monotherapy (31.7 %) or in combination with other antifungal agents. The superiority of AmB monotherapy or combination therapy compared to other therapeutic options against Mucorales infections was observed in a previously published study with solid organ transplantation (SOT) patients suffering from mucormycosis. This study concluded that AmB might influence overall survival. However, in other studies the choice of first line antifungal therapy did not significantly influence the survival rates of the patients [9, 10, 19]. In our study, the impact of AmB as monotherapy or combined with other antifungals on overall survival could not be estimated, because the number of patients taking the various antifungals with AmB was too small and a statistical comparison was not feasible. In addition, concomitant confounding factors hindered further analysis of the present data. On the other hand, in our study combined antifungal therapy plus surgery remained an independent factor for survival. Results from previous studies are rather controversial. One study showed that surgery in adult patients does not significantly influence overall survival, while other studies stressed that surgery alone improves outcome, especially in SOT patients [1, 9, 15, 19, 21]. We can hypothesize that in localized infections surgery improves outcome by hindering dissemination, while in severely sick patients, combination of antifungal drugs plus surgery significantly reduces the overall burden of disease.

In the present study, further independent risk factors for mortality were dissemination and HSCT. These results are in concordance with previous data [19]. Thus, age <12 months was also an independent risk factor for mortality in paediatric patients, which was not the case in our case series, although these differences may also be partially explained by the significant differences in the median age of the children [1, 9, 19]. The overall mortality rate was 33.3 %. As expected, the mortality rates presented great variability among different anatomic sites and underlying conditions. In particular, localized infection and no underlying conditions or trauma presented the highest survival rates, while disseminated infection and HSCT were associated with increased mortality rate.

Limiting factors of the present study were associated with the nature of its design that was based on the extraction and evaluation of data from two differently organized global registries. This has led to the collection and careful analysis only of variables/data with exactly the same characteristics, such as definitions, stratifications, data recorded. For that reason clinical presentation of the cases as well as epidemiological time trends of paediatric mucormycosis were not analysed in the present study. A correlation between the specific countries and the variables presented in the study could be very useful to detect possible differences among cases worldwide. It is probable that the number of mucormycosis cases in each country in the present study does not necessarily reflect the country-specific rates of the disease, but the awareness of the investigators to report the cases in the two databases. Additionally, several countries reported only one to two cases during the study period therefore a statistical analysis between the variables and the specific counties was not feasible. In an attempt to detect differences in pediatric mucormycosis cases between EU and non-EU countries our analysis showed that there was no statistical difference reported as far as age, causative pathogens and survival is concerned. This result should be interpreted with caution since the number of non-EU cases was small to draw conclusions. On the other hand, due to the rarity of the disease, previous data from the literature until now were mainly extracted from i) mixed adult-children population databases with a limited number of paediatric cases, ii) very small paediatric case series or iii) literature review analyses [1, 2, 9, 10, 12]. In that sense, this is a first time of prospectively collecting and analysing global contemporary cases of mucormycosis in children.

One of the major strengths of the present study is the “merging” of data in order to obtain a satisfactory pediatric sample. This is prerequisite in order to obtain results with sufficient statistical power and to draw conclusions for rare fungal diseases. Beyond the limitations mentioned, the present study increases the degree of awareness of this potentially fatal disease among physicians and offers further important information enriching our knowledge on epidemiology and current management of invasive mucormycosis in the paediatric setting from two international databases.

## Conclusions

This is an epidemiologic study analysing a large number of invasive mucormycosis contemporary cases in paediatric patients prospectively recorded in two international registry databases. Mucormycosis mainly affects children with malignancies or HSCT and presents as disseminated or pulmonary infection. Mortality is improved when active antifungal therapy and surgery are combined.

## Additional files

Additional file 1: List of Ethics Committees that approved data collection and use for zygomyco.net. (DOCX 160 kb)
Additional file 2: List of Ethics Committees that approved data collection and use for Fungiscope. (DOCX 197 kb)

## Abbreviations

ALL: Acute lymphoblastic leukaemia
AMB: Amphotericin B
AML: Acute myeloid leukaemia
CAS: Caspofungin
CRFs: Case report forms
DM: Diabetes mellitus
FLU: Fluconazole
HL: Hodgkin lymphoma
HSCT: Haematopoietic transplantation
IFD: Invasive fungal disease
IQR: Inter quartile range
MIC: Micafungin
OD: Odds ratio
PCR: Polymerase chain reaction
POS: Posaconazole
SLE: Systemic lupus erythematosus
SOT: Solid organ transplantation
spp: Species
SPSS: Statistical package for the social sciences
T-NHL: non-Hodgkin lymphoma
US: United States
