# Supplementary material for: Invasive mucormycosis in children: an epidemiologic study in European and non-European countries based on two registries
Source: BMC Infect Dis. 2016 Nov 10;16:667. doi: 10.1186/s12879-016-2005-1 (PMC5105268; doi:10.1186/s12879-016-2005-1)
Supplement: Additional file 1: — List of Ethics Committees that approved data collection and use for zygomyco.net. (DOCX 160 kb) [file 12879_2016_2005_MOESM1_ESM.docx]

**Additional file 1**. List of Ethics Committees that approved data collection and use for zygomyco.net.

| **Name** | **Affiliation** | **Ethics approval** |
| --- | --- | --- |
| Conny Lass-Flörl | Medizinische Universität Innsbruck, Innsbruck, Austria | Agreed w/o letter of approval |
| Jagdish Chander | Government Medical College Hospital , Chandigarh , India | Agreed w/o letter of approval |
| Monika Rolencova | University Hospital Brno, Brno, Czech Republic | Ethics approval |
| Zdenek Racil | University Hospital Brno, Brno, Czech Republic | Ethics approval |
| Vanda Chrenkova | Department of Department of Medical Microbiology, University Hospital Motol, Praha | Ethics approval |
| Fabianne Carlesse | Institute of Paediatric Oncology, Universidade Federal de São Paulo, Brazil | Agreed w/o letter of approval |
| Katrien Lagrou | National Reference Center for Mycosis, University Hospitals Leuven, Leuven, Belgium | Agreed w/o letter of approval |
| Livio Pagano | Università Cattolica del Sacro Cuore, Roma, Italy | Ethics approval |
| Sofiya Khostelidi | Metchnikov North-Western State Medical University, St Petersburg, Russia | Agreed w/o letter of approval |
| Nikolai Klimko | Metchnikov North-Western State Medical University, St Petersburg, Russia | Agreed w/o letter of approval |
| Bernard Dupont | Hopital Necker, 75015, Paris, France | Agreed w/o letter of approval |
