# Supplementary material for: Invasive mucormycosis in children: an epidemiologic study in European and non-European countries based on two registries
Source: BMC Infect Dis. 2016 Nov 10;16:667. doi: 10.1186/s12879-016-2005-1 (PMC5105268; doi:10.1186/s12879-016-2005-1)
Supplement: Additional file 2: — List of Ethics Committees that approved data collection and use for Fungiscope. (DOCX 197 kb) [file 12879_2016_2005_MOESM2_ESM.docx]

**Additional file 2**. List of Ethics Committees that approved data collection and use for Fungiscope.

| **Name** | **Affiliation** | **Ethics approval** |
| --- | --- | --- |
| Conny Lass-Flörl | Medizinische Universität Innsbruck, Innsbruck, Austria | Agreed w/o letter of approval |
| Werner Heinz | University Hospital of Würzburg, Würzburg, Germany | Agreed w/o letter of approval |
| Alberto Arencibia Núñez | Hematology and Immunology Institute, Havana, Cuba Hernan Henrique Aravena Hospital, La Frontera University, Temuco, Chile. | Agreed w/o letter of approval |
| Anupma Jyoti Kindo | Sri Ramachandra Medical College and Research Institute, Porur, Chennai, Tamil Nadu, India | Agreed w/o letter of approval |
| Monika Rolencova | University Hospital Brno, Brno, Czech Republic | Ethics approval |
| Zdenek Racil | University Hospital Brno, Brno, Czech Republic | Ethics approval |
| Vanda Chrenkova | Department of Department of Medical Microbiology, University Hospital Motol, Praha | Ethics approval |
| Petr Sedlacek | Department of Paediatric Haematology and Oncology, University Hospital Motol, Praha | Ethics approval |
| Julia Horakova | Department of Hematological Oncology and Bone Marrow Transplantation, Children´s University Hospital Bratislava, Slovakia | Ethics approval |
| Peter Mudry | Department od Pediatric Oncology, Masaryk University and University Hospital Brno | Ethics approval |
| Sofiya Khostelidi | Metchnikov North-Western State Medical University, St Petersburg, Russia | Agreed w/o letter of approval |
| Nikolai Klimko | Metchnikov North-Western State Medical University, St Petersburg, Russia | Agreed w/o letter of approval |
| Maria JGT Vehreschild | University Hospital of Cologne, Cologne, Germany | Agreed w/o letter of approval |
